# How Changes in Cash Transfers Can Affect Childbearing Among Low-Income Women: Evidence from the Finnish Basic Income Experiment

**DOI:** 10.1007/s10680-025-09735-9

**Published:** 2025-03-26

**Authors:** Miska Simanainen

**Affiliations:** 1 Department of Sociology, Stockholm University, Stockholm, Sweden; 2 Social Insurance Institution of Finland, Helsinki, Finland

**Keywords:** Childbearing, Cash transfers, Cash incentives, Basic income, Field experiment, Low-income women

## Abstract

The study analyses how a two-year guaranteed income program that provided a significant earnings supplement affected childbearing among previously unemployed women. Results from previous research indicate that cash transfers may increase fertility, for example, by compensating for the additional costs of childbearing and lost earnings opportunities. However, cash transfers may also induce incentives that lead to opposite fertility effects. The study provides new empirical evidence on the effect of cash transfers on childbearing by using register data from the Finnish basic income experiment conducted in 2017–2018. The intervention increased returns from employment relative to unemployment. As a result, it increased cash incentives to employment and other activities, such as studying, that compete with childbearing. The experiment offers a unique opportunity to study the causal effect of these changes on women’s childbearing decisions. According to the analysis, the experiment had a negative effect on the probability of having children among women who received basic income and a positive effect among women whose spouses received basic income. The findings suggest that while improvements in economic circumstances likely have a positive effect on childbearing, benefits conditional to working or other competing activities may have the opposite effect, at least in the short term. The findings emphasize the importance of considering the overall changes of income and cash incentives when reforming tax-benefit policies.

**Supplementary Information:**

The online version contains supplementary material available at 10.1007/s10680-025-09735-9.

## Introduction

The study aims to empirically investigate the role of cash transfers in the timing of having a child among low-income women. Several previous studies have tried to explore how changes in cash transfer policies affect family choices (e.g. Andersen et al., 2018; Azmat & González, 2010; Baughman & Dickert-Conlin, 2003, 2009; Berniell et al., 2020; Bitler et al., 2004; Cain & Wissoker, 1990; Cohen et al., 2013; González & Trommlerová, 2021; Groeneveld et al., 1980; Hart & Galloway, 2023; Hu, 2003; Moffitt, 1990; Rosenzweig, 1999; Weiss & Willis, 1997; Yonzan et al., 2020). Findings from the studies vary depending on the details of policy reforms and the contexts of policy implementation, indicating a complex relationship between income, cash transfers, incentives, and childbearing decisions.

During the last decades, guaranteed income programs and earnings supplements have gained popularity as alternative social policies to the mainstream tax-benefit systems to reduce poverty and provide equal life chances for individuals. In a welfare state context, these policies are assumed to increase individuals’ financial autonomy and employment opportunities and to remove the so-called welfare traps created by the distortions in the mainstream tax-benefit systems. So far, there has been only little evidence available on different social consequences of these policy alternatives. To contribute to the knowledge gap, this study examines the short-term fertility effects of a temporary guaranteed income program that provided a significant earnings supplement among low-income women.

In general, identifying the effects of social policies on demographic outcomes is challenging because family choices are greatly influenced by factors like social norms and personal preferences, which are difficult to observe. However, social policy experiments can provide a promising framework for contributing to the research. This article utilizes the study design and data from the Finnish basic income experiment, a randomized field experiment conducted in 2017–2018, to investigate the effects of changes in cash transfers on childbearing. The Finnish experiment offers an interesting case to explore because it tested a welfare alternative that improved the participants’ financial situation by providing an unconditional income guarantee. Results from previous research indicate that improvements in individuals’ and households’ financial circumstances may increase fertility (Bergsvik et al., 2021; Hart et al., 2024). In addition to providing a guaranteed minimum income, the Finnish experiment increased the participants’ cash returns from work significantly and, thus, created an incentive for participating women to postpone childbearing after the experimentation period.

In the experiment, a non-taxable cash payment (a basic income) equal to the net amount of the mainstream basic unemployment allowance, sickness allowance, or parental benefits was delivered to the participants unconditionally, regularly, and individually. The policy did not alter the level of income for most beneficiaries because basic income payments were deducted from primary social benefits. Instead, it increased cash returns from work because basic income payments were not tested against employment status or earnings.

By design, the experiment improved the economic resources of full-time workers, but also of students, and those who received no benefits from the mainstream cash transfer system. However, it barely affected the income of those who were taking care of a small child full time. Especially for women, childbearing is, at least partly and in a short time perspective, a life choice that competes with activities like working full time or studying (or perhaps pursuing some other personal life goals). Thus, this type of a policy may increase the opportunity cost of having a child and have a negative effect on childbearing among female participants in the short term.

The study design of the Finnish basic income experiment and the available register data provide a unique opportunity to assess the fertility effects of restructuring cash transfers among low-income women. The study design of a randomized field experiment surpasses the limitations of observational studies by producing an exogenous change in income and the incentive structures of the participants and by providing a control group against which to measure the changes that follow.

The study contributes to the knowledge of how changes in cash transfer policies can affect childbearing behaviour among the low-income population. This knowledge is vital for assessing policy alternatives and for avoiding unintended social outcomes when implementing the policies.

## Previous Research

Reforming cash transfer programs is often a trade-off between the potential benefits of the transfers and their costs. The costs include distortions that the programs and policies generate through their eligibility and calculation rules (Aizer et al., 2020). The potential distortions might have unintended effects on the behaviour and well-being of the individuals and families. As a result, fertility effects of cash transfers have received considerable attention in social research (Moffitt, 1992).

Cash transfers and their reforms can be designed in numerous different ways and there may be several simultaneous different mechanisms contributing to their fertility outcomes. For example, cash transfers can have *income effects* due to providing a general increase or decrease in income. Change in income may affect fertility directly independent of any other behavioural outcomes. On the other hand, cash transfers reforms can induce *incentive effects* by increasing returns from some behaviours like having more than one child, educating oneself, or working.

Some empirical studies have examined the effects of different types of conditional cash transfers on fertility in low-income countries. In several cases, conditional cash transfers have been designed to incentivize, for example, schooling instead of childbearing because, especially among young women, childbearing may reduce opportunities for education and increase the likelihood of poverty (see, e.g. Baird et al., 2011). For example, Gulemetova-Swan (2009) analysed the effects of the urban Oportunidades (Progresa) program, a nationwide antipoverty intervention in Mexico, on young women’s fertility decisions by using data from the 2002–2004 evaluation study. The program aims to improve education, health, and nutrition through conditional cash transfers and health education. In the program, cash payments are conditional on regularly attending high school and having mandatory health check-ups and educational health sessions. According to the study, young women (under 22 years old) living in the intervention areas significantly delayed having their first and second child.

Bastagli et al. (2019) reviewed the findings (published during 2000–2015) about the impact of different non-contributory cash transfers on individuals and households in low and middle-income countries. They concluded that the evidence points to non-contributory cash transfers having a significant negative impact on women’s fertility choices in general, but the exceptions to this rule are policies that link the levels of cash payments to the number of children (e.g. Stecklov et al., 2006).

In the high-income countries, cash transfer policies often aim to reduce income poverty or provide monetary compensation for those who are unemployed or temporarily outside the labour force, for example, when taking care of a child at home. In North American and European countries, the available studies on fertility outcomes of cash transfers are mostly non-experimental, and thus, the causal evidence on fertility outcomes is limited. One group of studies focuses on benefits that compensate for the costs of having a child. For example, González and Trommlerová (2021) studied the introduction and termination of a universal child benefit in Spain in 2007 and 2010, respectively. They found that the introduction of the policy led to a 3% increase in birth rates. The announcement of the cancellation led to a transitory increase in birth rates just before the benefit termination was implemented, driven by a short-term decrease in abortions. The actual cancellation of the policy led to a 6% decline in birth rates.

Incentives created by child-related benefits may also lead to unexpected outcomes. In the context of established parental leave schemes, Andersen et al. (2018) assessed the relationship between cash transfers and fertility in the case of a cash-for-care policy introduced in Norway in 1998. The policy provided a cash transfer to mothers who were taking care of a 13–36-month-old child at home. The authors compared the subsequent fertility behaviour of eligible and ineligible mothers over a four-year period and found that eligible mothers had a slower progression to second births and lower short-term fertility than the ineligible mothers.

Another line of research has focused on the effects of cash transfers that are not conditional to having a child. For example, Cowan and Douds (2022) analysed the impact of the Alaska Permanent Fund Dividend that has provided all Alaskan residents with a substantial annual cash payment since 1982. They examined the effects of the cash transfer on fertility among a large and diverse population receiving varying amounts of cash payments over time and found that the payments increased short-term fertility rates, particularly among socioeconomically disadvantaged populations. Their results indicated that receiving additional income may reduce economic constraints to having a child. Other studies have utilized lottery winners as quasi-experimental samples to study the effect of income and wealth on fertility (Bleakley & Ferrie, 2016; Bulman et al., 2022; Cesarini et al., 2023; Tsai et al., 2022). Consistent with the economic models of fertility (Becker, 1960, 1981; Willis, 1973), these studies document small but positive effects of wealth on fertility.

Some evidence on the effects of tax programs aiming to provide a guaranteed income has been available from the experiments testing a negative income tax system (NIT) in the US and Canada in the 1970s. In NIT, a minimum income level is provided by paying cash transfers to taxpayers who are earning below a certain income threshold. In the Gary Income Maintenance Experiment, having an income guarantee decreased fertility (Salkind & Haskins, 1982), while in the Manitoba Basic Annual Income Experiment the guaranteed income policy had a positive effect on childbearing (Dökmeci et al., 2023). Overall, however, studies on NIT experiments have found no impact on fertility (Marinescu, 2018).

More recent studies have utilized changes in the earned income tax credit system (EITC) in the USA as a quasi-experimental setup to study the fertility effects of taxation. According to available studies (e.g. Hoynes et al., 2015), EITC expansions in the USA during the 1990s did not affect overall fertility. Small, if any, reductions have been documented in the higher-order (three or more children) birth rates for White women in cases where expansions were targeted to families with children (Baughman & Dicker-Conlin, 2009). Other studies on EITC expansions (Duchovny, 2001) and on comparable tax reforms in other countries (e.g. for Spain, Azmat & González, 2010) have found similar results.

To conclude, the broader literature documents that the fertility elasticities with respect to changes in cash transfers are generally small (Moffitt, 1998). However, previous theoretical and empirical literature supports the expectation that cash transfers tied to childbearing, parental leaves, and the presence and number of children may lead to increases in fertility (see also Ang, 2015; Riphahn & Wiynk, 2017). On the other hand, economic reasoning suggests that the work-inducing aspect of earnings credits and supplements could lead to reductions in fertility due to an increase in the opportunity cost of the mother’s time. Previous empirical studies have not found strong evidence on the presence of the latter mechanism in childbearing decisions, but one may debate if the earlier studies have really been able to test the hypothesis in a controlled fashion. The Finnish basic income experiment creates a unique opportunity to contribute to the research and to test how changes in cash incentives may affect fertility among low-income women in high-income countries.

## The Basic Income Experiment

The main objective of the Finnish basic income experiment was to increase the employment of persons receiving basic unemployment benefits by providing them an additional temporary cash transfer program. The policy provided the participants with a guaranteed level of income and a significant earnings supplement when compared to the mainstream tax-benefit system. The experiment ran for two years from January 2017 to December 2018, taking place within the otherwise unaffected Finnish tax-benefit and social service system that represents a Nordic welfare state.

The Finnish experiment provided an unconditional monthly cash payment, a basic income, to the participating individuals for up to two years. In the experiment, the point of comparison is the mainstream welfare system without basic income, which provides income insurance for unemployment, sickness, parenthood, and retirement as well as additional cash assistance on the basis of means testing.

Persons aged 25–58 who received basic unemployment benefits in November 2016 were eligible for the experiment (target population). After applying specific exclusion criteria,^1^ the total target population of the experiment added up to 175,222 persons. The experiment was designed as a randomized controlled trial. To assess the effects of the basic income policy, primarily on economic behaviour, 2000 persons were assigned at random to a treatment group that was eligible for the basic income payments. The rest of the target population served as the control group. Persons in the target population had relatively low income and a relatively difficult position in the labour market: on average, they received €1900 in earnings and were paid unemployment benefits for 286 days in 2016, nearly 80% of them had completed secondary education at the most, and 16% had reported incapacity for work at the employment services (Verho et al., 2022).

The treatment group was paid an unconditional cash transfer of €560 on the second business day of each month. The amount was about equal to the net amount of minimum unemployment benefits and minimum parental and sickness allowances. The amount was deducted from other social benefits (for example, unemployment benefits, parental allowance, or sickness allowance) paid for the overlapping time period and considered as benefit income when calculating potential housing support and social assistance of the receiving household.

The basic income payments were non-taxable and no income or means-testing was applied. In addition, the personal income tax schedule remained the same for the treatment group and control group. This led to much higher work incentives in the treatment group than in the control group. For example, for a person earning up to €2000 per month from work and not receiving any means-tested benefits, the intervention would have increased incomes before taxes and work-related expenses by 28%. A microsimulation analysis that considers the actual recipiency of means-tested benefits and the study subjects’ spouses’ income estimated that, with monthly earnings of €2000, the net income of the participating household would have increased by almost one fourth, on average (Hämäläinen et al., 2020). For those who were eligible for unemployment benefits or other basic social benefits and who had no earnings, being in the basic income group mostly meant getting part of their benefit income more regularly than in the control group.

Persons in the basic income group continued to be eligible for all benefits for families with children with the same criteria as the control group, i.e. there were no changes made to maternity grants, child benefits, or child maintenance allowances. If the person received maternity/parental leave benefits, then basic income payments were deducted from the other benefits paid for the overlapping period. If the person received child home care allowance, the basic income payments were cancelled for the overlapping period.

The treatment group received the basic income payments for up to two years, as long as they did not move abroad for more than 30 days, get imprisoned, or start receiving a pension. The control group never received the basic income but continued to receive basic social benefits and welfare according to the usual eligibility rules. The experimental analysis of the experiment requires that the entire treatment group is compared with the control group (i.e. following intention-to-treat strategy) because these are the two groups that were randomly assigned and were, therefore, nearly identical in their observed and unobserved characteristics, except that one group was eligible for the basic income scheme.

## Theoretical Framework and the Study Hypotheses

The primary aim of the empirical part of the study is to examine (I) the average treatment effect^2^ of the Finnish basic income experiment on the participating women’s probability of giving birth. The outcome is defined as having at least one child during the follow-up period that extends to a maximum of 40 months from the beginning of the experiment. The measurement period starts in the beginning of the 9th month from the initiation of the experiment. Persons in the treatment group received information on being part of the experiment right before the experiment started, so births before the 9th month should not be affected by the experiment. Based on economic reasoning and previous research, one might expect that improvements in financial circumstances, i.e. a guaranteed income for all and additional income for those who found a job during the experiment, had a positive effect on fertility (*income effect*). However, a closer look at the cash incentives imposed by the basic income experiment gives reasons to expect that, for the participating women, also an opposing causal mechanism was present.

### Cash Incentives Changed Among Female Basic Income Recipients

In the experiment, basic income payments provided a regular, minimum level of income for the participants without conditions. However, the basic income payments also served as relatively generous monthly earnings supplements. The experiment increased work incentives significantly, leading to an increase of over 23% in the household disposable income if the participants’ monthly earnings were €2000 (Hämäläinen et al., 2020). On the other hand, as the basic income payments were deducted from other social benefits, there were no similar increases in the participants’ benefit incomes. For example, family benefits were basically not affected by the experiment, except the payment days were slightly changed compared to the mainstream system. In sum, the experiment increased the attractiveness of employment relative to unemployment. However, it also increased the opportunity cost of childbearing by increasing the monetary returns from employment but not from childbearing. Moreover, the gains from the program were available for only a limited period known by the participants, strengthening the incentive to postpone having a child (*incentive effect*).

Findings from the main evaluation study support the possibility that the hypothesized mechanism, i.e. women participants postponing childbearing in order to make use of the temporary earnings opportunity, may be present: During the second year of the experiment, positive effects on employment were found. On average, persons in the treatment group worked more than persons in the control group, measured both in the share of persons having employment days during a month and in the average number of days in employment during the entire year: During the first year, the employment rate increased from 8 to 18% in both study groups, but the estimated employment effect was not statistically significant (Verho, 2022). During the second year, the employment rate increased to 27% in the treatment group and to 25% in the control group, and the estimated employment effect was 6.6 days (8.6%). Among women, the estimated employment effect for the second year was 6.8 days, statistically significant at 10% significance level (Online Appendix in Verho, 2022). To have a crude scale for the magnitude of the expected fertility effect, a 2-percentage-point increase in employment rate would translate to a maximum of 2-percentage-point decrease in the share of women giving birth to a child during the experiment.

The experiment also increased incentives to some activities outside the labour force. Basic income payments were higher than the study grant, and they did not reduce the maximum number of available student grant months. Therefore, the basic income payments increased the incentives for participating in education during the experiment. Other possible activities that may compete with childbearing include, for example, participating in volunteer or any unpaid work full time or perhaps pursuing some other personal life goals. In the basic income group, persons faced no behavioural requirements in order to receive a basic income, which may have opened up new financial opportunities to otherwise unavailable life choices.

The magnitude of the potential negative effect of the incentives may be hypothesized to be age dependent. Older women have a higher risk of not being able to get pregnant, and thus, the expected cost of postponing childbearing may be larger for them (Schmidt et al., 2012). Moreover, the variation in the effect along immigration background is of interest because, on average, immigrant women may follow different childbearing and labour participation patterns than the native population. Finally, decision-making regarding having the first and subsequent children may follow different logics, motivating subgroup analyses for women with and without dependent children.

### Household Income Increased Among Women Whose Spouses Received Basic Income

To complement the primary aim, the secondary aim of the study is to analyse (II) the average treatment effect of the Finnish basic income experiment on the probability of giving birth among women whose spouses participated in the experiment. In addition to providing a minimum level of income for the spouses, the experiment induced an increase in the household income of these women if their participating spouses found a job during the experiment (*income effect*). Verho et al. (2022) document an increase of 6–7% in the total annual income for all the participants of the experiment, on average.^3^ However, the women whose spouses participated in the experiment were themselves not exposed to the increased work incentives (or incentives to other activities competing with childbearing) because their behaviour did not affect the recipiency of basic income payments. Taking women whose spouses received basic income as a separate study population offers a setting similar to studies analysing the fertility effects of a general income increase (e.g. Bleakley & Ferrie, 2016; Bulman et al., 2022; Cesarini et al., 2023; Cowan & Douds, 2022; Hart & Galloway, 2023; Tsai et al., 2022; see also Bergsvik et al., 2021; Hart et al., 2024) and, thus, provides an interesting point of comparison for the main analysis. Based on the arguments presented in this section, Table 1 summarizes the study questions, assumed causal mechanisms, and empirical hypotheses of the study regarding the potential fertility effects of the Finnish basic income experiment.

**Table 1:** Summary of the study questions and hypotheses

Study question	Causal mechanisms	Empirical hypotheses
How the basic income experiment affected childbearing among **female participants**	Female participants respond to a temporary earnings opportunity (or other life opportunity) by **postponing** childbearing	A **negative** *incentive effect* on the cumulative probability of having a child during the experiment that levels off after the experiment
	Increased personal income and financial security **compensate** for the additional costs and the lost earnings opportunity due to having a child	A lasting **positive** *income effect* on the probability of having a child (anticipation and level effect)
How the basic income experiment affected childbearing among **women whose spouses participated in the experiment**	Increased household income and financial security **compensate** for the additional costs and the lost earnings opportunity due to having a child	A lasting **positive** *income effect* on the probability of having a child (anticipation and level effect)

## Data

In the study, administrative registers were used to collect individual-level data before the time of random assignment as well as during and after the experimentation period. The original target population of the Finnish basic income experiment included 175,222 persons, i.e. almost all persons who received basic unemployment benefits in November 2016. To set up the experiment, 2000 persons from the target population were allocated to the treatment group and 173,222 to the control group. As the primary aim of this study was to examine (I) the average treatment effect of the basic income experiment on the participating women’s probability of giving birth, a further inclusion criterion was implemented: only women aged below 40 in the beginning of the experiment were selected from the original treatment and control group. The exclusion of men and older age groups resulted in a study population comprising 462 women in the treatment group and 41,012 women in the control group.

The outcome variable of the study, i.e. the time of having a child, was derived from the Benefit Register of the Social Insurance Institution by collecting information on children’s birth dates from the records of maternity grants received by the study subjects. A maternity grant that can be taken as a maternity package or a tax-free lump sum of €170 is claimed each time a woman has a new child.

Every person in the study population was followed from the registers starting from the treatment assignment in December 2016 until the end of the available data. Data on maternity grants extend to February 2020 and, in practice, include information on births until April 2020, i.e. 40 months from the beginning of the experiment. With these data, a monthly indicator of having a child during the 40-month follow-up period was constructed. For the indicator, multiple births were calculated as single events, i.e. a study subject can have only one birth per month.

In addition to the main study outcome, information on several baseline characteristics (measured prior to the experimentation period) including allocation to the treatment group, age, marital status, cohabitation, number of dependent children, native language, place of residence, and taxable income was collected from the Basic Income Experiment Register, Benefit Register of the Social Insurance Institution, and Tax Register. Treatment status indicates the study group to which the study person belongs, i.e. the basic income group or control group. Marital status is categorized as never married, married, divorced, or widowed, and the same information is included for registered partnerships (same-sex couples). Cohabitation is defined as cohabiting without marriage, and dependent children are defined as 0–17-year-old children of a legal guardian.^4^ Native language is defined as a person’s officially registered first language. Place of residence is categorized into municipality groups (urban, semi-urban, rural) according to the municipality’s degree of urbanization, i.e. according to the proportion of population living in urban settlements and the size of the population in the largest urban settlement in 2016 (Statistics Finland, 2015). Taxable income measures the total annual earned and capital income (including taxable social benefits) before tax deductions. The original source for demographic variables in the Benefit Register of the Social Insurance Institution (age, marital status, native language, and the place of residence) is Population Register. The baseline variables are used to describe the study population and to divide it into subgroups for the analysis of effect heterogeneity.

For the secondary aim of the study, i.e. to analyse (II) the average treatment effect of the basic income experiment on the probability of giving birth among women whose spouses participated in the experiment, an additional study population was formed, and the same outcome and background variables were collected when available. The additional study population includes 20–39-year-old women who had a spouse in the target population of the basic income experiment right before the experiment started. Spouses are defined as spouses in marriage or registered partners as recorded in the Population Register data in December 2016, or as spouses in the General Housing Allowance Register (i.e. cohabiting partners). The additional study population included 126 women in the treatment group and 10,749 in the control group.

## Descriptive Statistics

The baseline characteristics of the persons in the treatment and control groups of the main study population (female basic income recipients) are presented in Table 2. At baseline, about two fifths were married or in a registered partnership, 46% had never been married, and about one sixth were cohabiting without marriage. About two fifths had no dependent children. Every third woman had other than Finnish or Swedish (official domestic languages in Finland) as their native language, and about four fifths of the persons in the study population lived in urban municipalities. Average taxable income during the year preceding the experiment was less than €11,000 in both of the study groups.

**Table 2:** Baseline characteristics of the female basic income recipients, separately for the basic income group and control group

Baseline characteristic	Basic income group	Control group	p-value
Age			0.199
25–29 years (%)	30.7	34.7	
30–34 years (%)	37.2	34.6	
35–39 years (%)	32.0	30.7	
Marital status			0.876
Never married (%)	45.9	45.6	
Married^a^ (%)	41.3	40.2	
Divorced or widowed (%)	10.2	11.3	
No information (%)	2.6	2.9	
Cohabiting (%)	16.5	16.3	0.950
Number of dependent child (%)			0.776
No children (%)	42.4	40.9	
One child (%)	21.9	21.0	
Two children (%)	19.9	21.6	
Three or more children (%)	15.8	16.4	
Foreign language (%)	31.8	31.3	0.977
Municipality group			0.417
Urban (%)	82.3	81.0	
Semi-urban (%)	10.8	10.4	
Rural (%)	6.5	8.3	
No information (%)	0.4	0.3	
Taxable income^b^ (€)	10,693	10,879	0.357
N	462	41,012	

Fisher’s exact test applied to categorical and Welch two sample t-test applied to continuous background characteristics. Age, marital status, native language, and municipality group on Dec 31, 2016. Cohabitation and number of dependent children in Nov 2016

^a^Including persons in registered partnership

^b^Average taxable income (earnings, capital income and taxable social benefits) for the entire year 2016

Table 2 shows that the random allocation produced study groups of female basic income recipients that were mostly similar in their examined background characteristics. Statistically significant differences were not found between the treatment and control group for any of the examined background characteristics. In order to adjust for the differences in the background characteristics between the research groups, age and a number of other baseline covariates that predict fertility behaviour are included in the additional estimations provided in the Supplement.

The baseline characteristics of the persons in the treatment and control groups of the additional study population (the women whose spouses received basic income) are presented in Table 3. The additional study population is somewhat different compared to the study population of the main analysis. It also includes 20–24-year-old women.^5^ At baseline, more than two thirds were married or in a registered partnership, and the rest of the women were cohabiting without marriage. About half of the women had other than an official domestic language as their native language, and almost 90% of the persons in the additional study population lived in urban municipalities.

**Table 3:** Baseline characteristics of the women whose spouses received basic income, separately for the basic income group and control group

Baseline characteristic	Basic income group	Control group	p-value
Age			0.352
20–24 years (%)	16.7	12.9	
25–29 years (%)	30.2	30.8	
30–34 years (%)	25.4	31.3	
35–39 years (%)	27.8	25.1	
Marital status			0.510
Never married (%)	24.6	30.0	
Married^a^ (%)	69.8	64.6	
Divorced or widowed (%)	4.8	3.9	
No information (%)	0.8	1.6	
Cohabiting (%)	30.2	35.4	0.225
Foreign language (%)	54.8	43.1	0.011*
Municipality group			0.354
Urban (%)	88.1	87.3	
Semi-urban (%)	5.6	7.3	
Rural (%)	5.6	5.3	
No information (%)	0.8	0.2	
N	126	10,749	

Fisher’s exact test applied to categorical background characteristics. Age, marital status, native language, and municipality group on Dec 31, 2016. Cohabitation in Dec 2016

^a^Including persons in registered partnership

^*^p < 0.05

The random allocation produced study groups of women whose spouses received basic income that are mostly similar in their examined background characteristics, except the treated in the additional study population were more likely to have other than Finnish or Swedish as their native language compared to the controls (significant at 5% significance level).

To enable causal inference about the average treatment effect of the intervention, the original experimental design of the Finnish basic income experiment is exploited. The fertility outcomes of women in the entire treatment group are compared to those in the control group, both among female basic income recipients (main population) and among women whose spouses received basic income (additional population). Because persons in the target population of the experiment were assigned to the treatment group or to the control group at random, the groups should be similar both in their observed and unobserved characteristics, such as preferences and motivations. Thus, any differences in the probability of having children between the study groups can be attributed to the intervention. Being in a basic income system or in the mainstream tax-benefit system is the only characteristic that differs systematically between the study groups.

## Estimation Method

The average treatment effect of being selected into the basic income system for two years on fertility outcomes was estimated using the following linear regression equation and the ordinary least squares estimator: *Y*_*i*_ = a + b × *T*_*i*_ + Sum(c_*k*_ × *X*_*k*_) + *e*_*i*_, where *i* is a unique person in the study, *Y* is the dependent variable, such as having a child during a specific follow-up period, *T* is the indicator of the person being assigned to the treatment group, *X*_*k*_ is the *k*^th^ baseline characteristic included as a control variable, and *e* is the normally distributed error term. Coefficient b represents the effect of being selected into the basic income system for two years on the dependent variable, and c_*k*_ is the average contribution of the *k*^th^ baseline covariate on the dependent variable. Estimations in Results section are based on a simple linear regression equation that includes only the treatment indicator as a predictor. Estimations based on multiple linear regression equations including age and other covariates as predictors are reported in the Supplement. Statistical inference is conducted using two-tailed t-test and setting the null hypothesis to no effect. Standard errors are heteroskedasticity robust (HC1). The results are tested to be consistent with results derived by using logistic regression or time-to-event analysis.

The main analysis is performed by estimating the regression model above separately for the following outcome variables: having at least one birth between month 9 and (I) month 16, (II) month 24 (the end of the experimentation period), (III) month 32, and (IV) month 40 (the end of the follow-up). Since the groups are assigned randomly and the fertility outcome is assumed to result from the decision to conceive post-assignment, births are calculated from the ninth month onwards. In addition to the main analysis, an examination of effect heterogeneity among women who participated in the experiment is conducted for the four follow-up periods (I, II, IIII, and IV) by estimating the average treatment effect separately for subgroups formed according to age, marital status and cohabitation, having dependent children, native language, and urbanization level of the municipality of residence. Due to low number of observations (*N*_treated_ = 126) effect heterogeneity is not analysed for the additional sample of women whose spouses participated in the experiment.

## Results: Fertility Effects of the Finnish Basic Income Experiment

### Effects on Female Basic Income Recipients

**Fig. 1:**
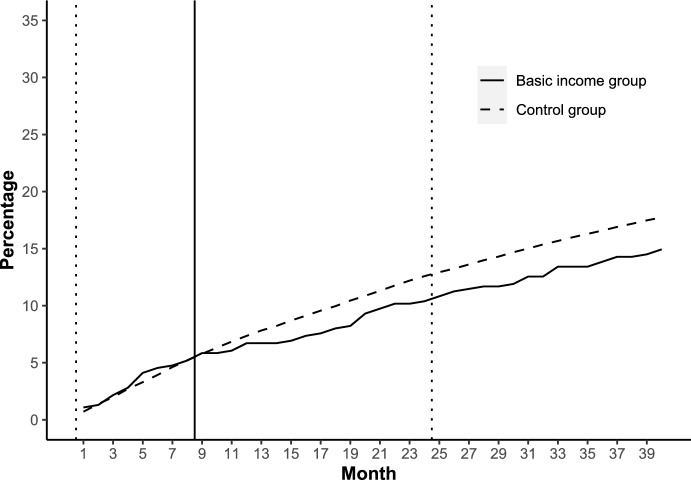
Cumulative share of women who had at least one child after the initiation of the experiment; 25–39-year-old women who participated in the experiment, separately for basic income group and control group; months 1–40

Calculation of births during the 40-month follow-up starting from the beginning of the experiment resulted in 77 births for 69 women giving birth in the treatment group (N = 462) and 8060 births for 7272 women giving birth in the control group (N = 41,012). Figure 1 shows the cumulative share of persons in the main study population having at least one birth after the initiation of the experiment, month by month, and separately for the basic income group and control group. During the first eight follow-up months of the experiment, no lasting difference in the cumulative share of persons having at least one birth is observed. After month 9, a gradually increasing difference between the groups emerges, resulting in a smaller cumulative share of women in the treatment group having at least one birth after treatment assignment than in the control group. The difference does not diminish after the experimentation period ends; instead, it lasts and even increases a bit throughout the rest of the follow-up period from month 24 to month 40.

Table 4 summarizes the average treatment effect of the intervention on having at least one birth between month 9 and month 16, 24 (the end of the experiment), 32, and 40 (the end of the follow-up).^6^ By month 16, 4.0% of the persons in the control group had given birth to a child. In the treatment group, the cumulative share is 1.8 percentage points (45.3%) lower, and the difference is statistically significant at 1% significance level. At the end of the experiment (month 24), 7.8% of the persons in the control group had given birth to a child, while in the treatment group, the cumulative share is 1.9 percentage points (24.8%) lower (statistically significant at 10% significance level). At the end of the follow-up (month 40), 13.6% of the persons in the control group had given birth to at least one child, and the estimated effect of the experiment is -2.4 percentage points (-17.4%), but not statistically significant at 10% significance level. Adjusting for age and other demographic covariates decreases the effect magnitudes slightly (see Table S1 in the Supplement).

**Table 4:** Effect of the basic income experiment on childbearing among low-income women; mean probability in the control group and absolute and relative difference with basic income during different follow-up periods

Outcomes	Mean probability in control group	Risk difference with basic income	Risk ratio with basic income	*p*-value
At least one birth since month 1				
By month 8	0.052	− 0.000	0.996	0.984
At least one birth since month 9				
By month 16	0.040	− 0.018	0.547	0.009**
By month 24	0.078	− 0.019	0.752	0.080^†^
By month 32	0.109	− 0.022	0.794	0.089^†^
By month 40	0.136	− 0.024	0.826	0.110

Two-tailed t-test applied to differences between the means of outcomes of basic income group and control group. Null hypothesis is P(Y = 1)_treat_ = P(Y = 1)_control_

^†^*p* < .10; **p < .01

About 12.7% of the persons in the control group and 10.6% in the treatment group had one birth, and about 0.9% of the persons in the control group and 0.6% in the treatment group had two or more births during the entire follow-up period from month 9 to month 40. A non-experimental comparison of the study groups shows that women in the control group who gave birth during the follow-up period (months 9–40) had it, on average, 23.1 months after the beginning of the experiment, while the women in the treatment group had it 1.2 months later, on average. The difference in the average month of having a birth is not statistically significant.

Table 5 reports the average treatment effects of the experiment by month 16 and by month 24 (the end of the experiment) among different subgroups of the study population, i.e. according to age, having or not having dependent children, native language, and municipality group. All effect estimates are negative, except for the 30–34-year-olds by month 24 and for those without dependent children by month 24. In addition, the magnitude of the two positive effect estimates is in substantial terms close to zero (0.5 percentage points and 0.2 percentage points, respectively).

**Table 5:** Effect of the basic income experiment on childbearing among subgroups of low-income women; mean probability in the control group (C) and absolute difference (Diff) with basic income by month 16 and 24 (end of the experiment)

Baseline subgroup	Months 9–16			Months 9–24			N of treated
	C	Diff	*p*-value	C	Diff	*p*-value	
Age							
25–29 years	0.049	− 0.028	0.021*	0.099	− 0.042	0.030*	142
30–34 years	0.041	− 0.006	0.651	0.082	0.005	0.805	172
35–39 years	0.027	− 0.020	0.004**	0.049	− 0.022	0.101	148
Married or cohabiting							
Yes	0.051	− 0.028	0.002**	0.099	− 0.028	0.077^†^	267
No	0.025	− 0.004	0.672	0.050	− 0.009	0.545	195
Having dependent children							
Yes	0.044	− 0.021	0.020*	0.087	− 0.035	0.012*	266
No	0.033	− 0.013	0.210	0.064	0.002	0.897	196
Native language							
Domestic	0.030	− 0.015	0.040*	0.059	− 0.021	0.050*	315
Foreign	0.060	− 0.026	0.090^†^	0.118	− 0.016	0.533	147
Municipality group^a^							
Urban	0.040	− 0.017	0.036*	0.078	− 0.010	0.463	380
Semi-urban–rural	0.037	− 0.025	0.050*	0.077	− 0.065	0.000***	80

Two-tailed t-test applied to differences between the means of outcomes of basic income group and control group. Null hypothesis is P(Y = 1)_treat_ = P(Y = 1)_control_

^a^Persons with no information on municipality group are excluded

^†^*p* < 0.10; *p < 0.05; **p < 0.01; ***p < 0.001

The magnitude of the effect estimate is clearly larger for women who are married or cohabiting at baseline (− 2.8 percentage points both by month 16 and month 24) compared to women who are not married and not cohabiting (− 0.4 percentage points by month 16 and 0.9 percentage points by month 24). Between age groups, the largest absolute effect magnitudes are estimated for the youngest age group (25–29-year-old women): − 2.8 percentage points by month 16 and − 4.2 percentage points by month 24. In addition, persons with dependent children are more responsive than persons without dependent children. Among women with children, the probability of having given birth by the end of the experiment is 3.5 percentage points lower in the treatment group than in the control group while among women without children no difference between the study groups is found at the end of the experiment. Regarding the women’s native language, there is no clear pattern between the effect estimates by month 16 and month 24. However, by the end of the experiment, the negative effect seems to be larger for women with a domestic language than for women with a foreign language. Finally, women residing in semi-urban or rural areas stand out as having a large negative effect of − 6.5 percentage points (statistically significant at 0.1% significance level) by the end of the experiment. The point estimate is, however, rather unstable due to low number of persons (*N*_treated_ = 80). The results from the subgroup analyses regarding effect heterogeneity should be interpreted with caution as the estimates are quite unprecise: the 95% confidence intervals overlap each other within all subgroup divisions.

### Effects on Women Whose Spouses Received Basic Income

**Fig. 2:**
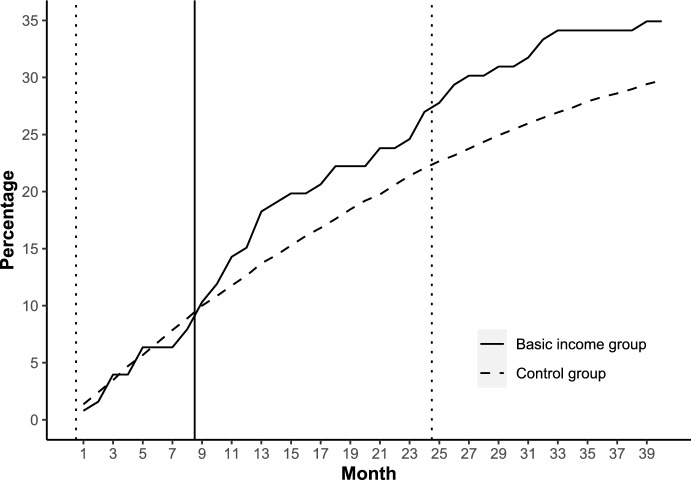
Cumulative share of women who had at least one child after the initiation of the experiment; 20–39-year-old women whose spouses participated in the experiment, separately for basic income group and control group; months 1–40

For women whose spouses received basic income, calculation of births during the 40-month follow-up resulted in 53 births for 44 women giving birth in the treatment group (*N* = 126) and 3600 births for 3197 women giving birth in the control group (*N* = 10,749). Figure 2 shows that during the first eight follow-up months, no lasting difference in the cumulative share of persons having at least one birth is observed. At month 9, a gradually increasing difference between the groups emerges, resulting in a larger cumulative share of women in the treatment group having at least one birth after treatment assignment compared to women in the control group, and the difference lasts and even increases throughout the rest of the follow-up period.

Table 6 summarizes the average treatment effect of the intervention on having at least one birth among women whose spouses received basic income.^7^ The effects are shown per follow-up period. By month 16, 7.3% of the persons in the control group had given birth to a child. In the treatment group, the cumulative share is 4.6 percentage points (62.0%) higher, but the difference is not statistically significant. By the end of the experiment (month 24), 14.1% of the persons in the control group had given birth to a child, while in the treatment group, the cumulative share is 6.6 percentage points (46.7%) higher (statistically significant at 10% significance level). The difference in the cumulative share increases by month 32 (8.6 percentage points, 45.2%, statistically significant at 5% significance level) after which it decreases. By the end of the follow-up (month 40), 23.0% of the persons in the control group had given birth to at least one child, and the estimated effect of the experiment is 7.1 percentage points (31.0%), statistically significant at 10% significance level.^8^ Adjusting for age and other demographic covariates decreases the effect magnitudes somewhat more than for women who participated in the experiment (see Table S2 in the Supplement).

**Table 6:** Effect of the basic income experiment on childbearing among women whose spouses participated in the experiment; mean probability in the control group and absolute and relative difference with basic income during different follow-up periods

Outcomes	Mean probability in control group	Risk difference with basic income	Risk ratio with basic income	*p*-value
At least one birth since month 1				
By month 8	0.089	− 0.009	0.893	0.700
At least one birth since month 9				
By month 16	0.073	0.046	1.620	0.116
By month 24	0.141	0.066	1.467	0.070^†^
By month 32	0.191	0.086	1.452	0.031*
By month 40	0.230	0.071	1.310	0.082^†^

Two-tailed t-test applied to differences between the means of outcomes of basic income group and control group. Null hypothesis is P(Y = 1)_treat_ = P(Y = 1)_control_

^†^*p* < 0.10; *p < 0.05

## Discussion and Conclusions

The results of the study show that a temporary guaranteed income program that offers a significant earnings supplement may have a negative effect on the probability of giving birth among low-income women in the short term. The Finnish basic income experiment, which provided additional income especially for the employed and also for students and persons active outside the labour force but not for persons having a child, had a statistically significant (at 1% significance level) effect of − 1.8 percentage points (− 45.3%) on the cumulative probability of giving birth by month 16 of the two-year experiment. The cumulative share of persons having a birth in the treatment group compared to the control group diverged 10 months after the beginning of the experiment. The cumulative share of persons having at least one child since month 9 remained lower in the treatment group throughout the entire follow-up period of 40 months (statistically significant at 5% significance level from month 14 to month 19, see Figure S1 in the Supplement).

The analysis of effect heterogeneity suggests that 25–29-year-old women were more responsive to the intervention than 30–34-year-old women who basically were unaffected. The finding is consistent with risk aversion regarding the probability of not being able to have a child that increases with age. The youngest age group has more time to catch up the fertility levels of the controls later, since nowadays, 30–34-year-old women represent the prime reproductive group in Finland. However, women aged 35–39 had an equally large relative effect on the probability of giving birth during the experiment as the youngest age group which may eventually turn out to be a long-lasting outcome.

In addition, women who had dependent children were more responsive than those without dependent children. In other words, the experiment seems to have resulted in the postponement of second or later births rather than first births in the short term. This observation resonates with the findings from the main evaluation study documenting the largest employment effects among participants who already had children (Verho et al., 2022).

The study was motivated by the expectation that the work-inducing aspect of earnings credits and supplements might lead to reductions in fertility due to an increase in the opportunity cost of the mother’s time. The findings suggest that a temporary cash transfer program that incentivizes employment in relation to childbearing may indeed decrease fertility among low-income women, at least in the short term. Similar causal mechanisms may also play a role in other policy reforms that incentivize any activities that compete with childbearing. Even if fertility elasticities with respect to cash transfer reforms may be generally small (Moffitt, 1998), optimization regarding the time of having a child and earnings opportunities seem to matter.

In addition to employment, also other competing activities outside the labour force may have a role in explaining the negative fertility effects found. In the Finnish experiment, basic income payments were higher than the student grant, and they did not reduce the maximum number of student grant months available for the participants. This increased incentives to educate oneself during the experiment and perhaps fostered another competing activity for childbearing. In general, the participants of the experiment faced no behavioural requirements if they did not claim for the mainstream benefits and instead decided to cope with the basic income payments. This may have opened up new financial opportunities for different life choices that compete with the choice of having a child in the short term.

Contrasting with the main analysis, the results for women whose spouses received basic income show evidence on a positive fertility effect that spans over the experimentation period. The finding suggests that, among low-income women, an increase in household income may increase fertility, as documented by other studies conducted in different policy contexts (e.g. Bleakley & Ferrie, 2016; Bulman et al., 2022; Cesarini et al., 2023; Cowan & Douds, 2022; Hart & Galloway, 2023; Tsai et al., 2022; see also Bergsvik et al., 2021; Hart et al., 2024) and expected by the economic models of fertility (Becker, 1960, 1981; Willis, 1973). The main limitation of the study in understanding the mechanism behind the positive fertility effects is that we cannot separate the potential effect of the spouses having an unconditional income guarantee from the genuine income effect.

The long-term fertility effects of the basic income experiment may be quite different. For example, preferring employment to childbearing in the short run may increase women’s economic buffers against losses of earnings due to childbearing later. By lowering the potential economic obstacle of having a child this causal chain could eventually lead to a positive overall fertility effect among the participating women. Previous studies have indeed indicated that while unemployment and job insecurities may decrease fertility, the long-term effects of employment may actually be positive, especially in the case of having the first child (e.g. Alderotti et al., 2021; Del Bono et al., 2012; Hofman et al., 2017; Huttunen & Kellokumpu, 2016; Kreyenfeld & Andersson, 2014). So far, however, the findings of the study are in contrast with the aforementioned studies and the original empirical hypothesis: The cumulative share of women giving birth in the treatment group remained at the lower level compared to the control group even by 16 months after the two-year experiment had ended.

The effects of a temporary policy may also be quite different compared to a permanent policy that provides earnings supplements for women also after giving birth and after the temporary period of caring for a small child at home has ended. A longer-lasting policy would likely induce smaller incentives to postpone childbearing compared to a temporary experiment. Taking different perspective, timing of policy programs may offer a powerful policy tool to effectively direct social behaviour.

To conclude, income and cash incentives seem to play a notable role in the fertility decisions of low-income women, and, thus, potential fertility effects are worth considering when reforming cash transfer policies. As Andersen et al. (2018) point out, policy changes may lead to changes in fertility behaviour, for example, by inducing changes in the complex incentive structures. Incentives themselves are determined both by the individual policies (here, especially the temporary nature of the experiment) and the wider policy framework (here, the parental benefit system that remains mostly unaffected by the reform). For example, increasing returns from work in relation to unemployment by introducing temporary earnings supplements may lead to unintended fertility outcomes if no parallel reforms are made to parental benefits. In addition, individually targeted policies may affect the behaviour of other household members differently–making the task of designing policies with intended outcomes even more complicated.

## Supplementary Information

Below is the link to the electronic supplementary material.

Supplementary file1 (DOCX 712 KB)

## Data Availability

Access to data that support the findings of this study was authorized by permissions from the officials that administer the registers. As a general rule, legal restrictions in Finland prevent the public sharing of sensitive pseudonymized data. In addition, the researchers’ permissions from the data providers do not allow data sharing. In principle, the data are available from the Social Insurance Institution of Finland and Finnish Tax Administration, but restrictions apply to the availability of these data, which were used under license for the current study.

## Footnotes

1 Persons were excluded if they, most importantly, were laid off, received child home care allowance, received pension, or lived outside Finland at the time of defining the target population (Finland, 2016).

2 The average treatment effect is interpreted as the difference in the probability of having a child that would be observed if everyone in the study population were in the basic income group versus if everyone in the study population were in the mainstream tax-benefit system, i.e. equivalent to the average of all individual risk differences under the two systems.

3 Including taxable market income, taxable social benefits, basic income and non-taxable social support.

4 Information on dependent children is used as a proxy of having had previous children during a longer time period. Because of the legal base, the proxy does not exactly measure the number of biological or adopted children which is not as such available in the data.

5 The original target population of the basic income experiment consisted of 25–58-year-old previous basic unemployment benefit recipients, but there were no restrictions applied to participants’ spouses.

6 See also Figure S1 in the Supplement.

7 See also Figure S2 in the Supplement.

8 Conducting the estimations without 20–24-year-old women, i.e. having the same age range as for the main study population, reduces the magnitude of the effect estimates and their statistical significance slightly, but the overall conclusions remain the same as when including the youngest age group in the analysis.
