# Supplementary material for: How Changes in Cash Transfers Can Affect Childbearing Among Low-Income Women: Evidence from the Finnish Basic Income Experiment
Source: Eur J Popul. 2025 Mar 26;41(1):9. doi: 10.1007/s10680-025-09735-9 (PMC11947399; doi:10.1007/s10680-025-09735-9)
Supplement: Supplementary file 1 — Supplementary file1 (DOCX 712 KB) [file 10680_2025_9735_MOESM1_ESM.docx]

Supplement

**Table S1** Effect of the basic income experiment on childbearing among low-income women; unadjusted and adjusted absolute difference in the mean probability with basic income

| Outcome | Un adjusted risk difference with basic income | p-value | Adjusted to age only^a^ | p-value | Adjusted to age and other covariates^b^ | p-value |
| --- | --- | --- | --- | --- | --- | --- |
| At least one birth since month 1 |  |  |  |  |  |  |
| By month 8 | -.000 | .984 | .000 | .981 | -.000 | .984 |
| At least one birth since month 9 |  |  |  |  |  |  |
| By month 16 | -.018 | .009** | -.017 | .012* | -.018 | .010* |
| By month 24 | -.019 | .080† | -.018 | .098† | -.018 | .092† |
| By month 32 | -.022 | .089† | -.021 | .111 | -.021 | .103 |
| By month 40 | -.024 | .110 | -.022 | .141 | -.022 | .125 |

*Notes:* Two-tailed t-test applied to differences between the means of outcomes of basic income group and control group. Null hypothesis is P(Y=1)_treat_=P(Y=1)_control_.

^a^ Binary control variables for 5-year age groups (25–29/30–34/35–39) at baseline.

^b^ Binary control variables for 5-year age groups, native language (foreign/domestic), number of dependent children (0/1/2/3 or more), marital status (married/other), and cohabitation at baseline.

†*p* < .10; **p* < .05; ***p* < .01

**Table S2** Effect of the basic income experiment on childbearing among women whose spouses participated in the experiment; unadjusted and adjusted absolute difference in the mean probability with basic income

| Outcome | Un adjusted risk difference with basic income | p-value | Adjusted to age only^a^ | p-value | Adjusted to age and other covariates^b^ | p-value |
| --- | --- | --- | --- | --- | --- | --- |
| At least one birth since month 1 |  |  |  |  |  |  |
| By month 8 | -.009 | .696 | -.010 | .688 | -.015 | .523 |
| At least one birth since month 9 |  |  |  |  |  |  |
| By month 16 | .046 | .116 | .046 | .114 | .040 | .169 |
| By month 24 | .066 | .070† | .066 | .066† | .055 | .125 |
| By month 32 | .086 | .031* | .088 | .029* | .073 | .062† |
| By month 40 | .071 | .082† | .072 | .077† | .056 | .162 |

*Notes:* Two-tailed t-test applied to differences between the means of outcomes of basic income group and control group. Null hypothesis is P(Y=1)_treat_=P(Y=1)_control_.

^a^ Binary control variables for 5-year age groups (25–29/30–34/35–39) at baseline.

^b^ Binary control variables for 5-year age groups, native language (foreign/domestic), and marital status (married/other) at baseline.

†*p* < .10; **p* < .05


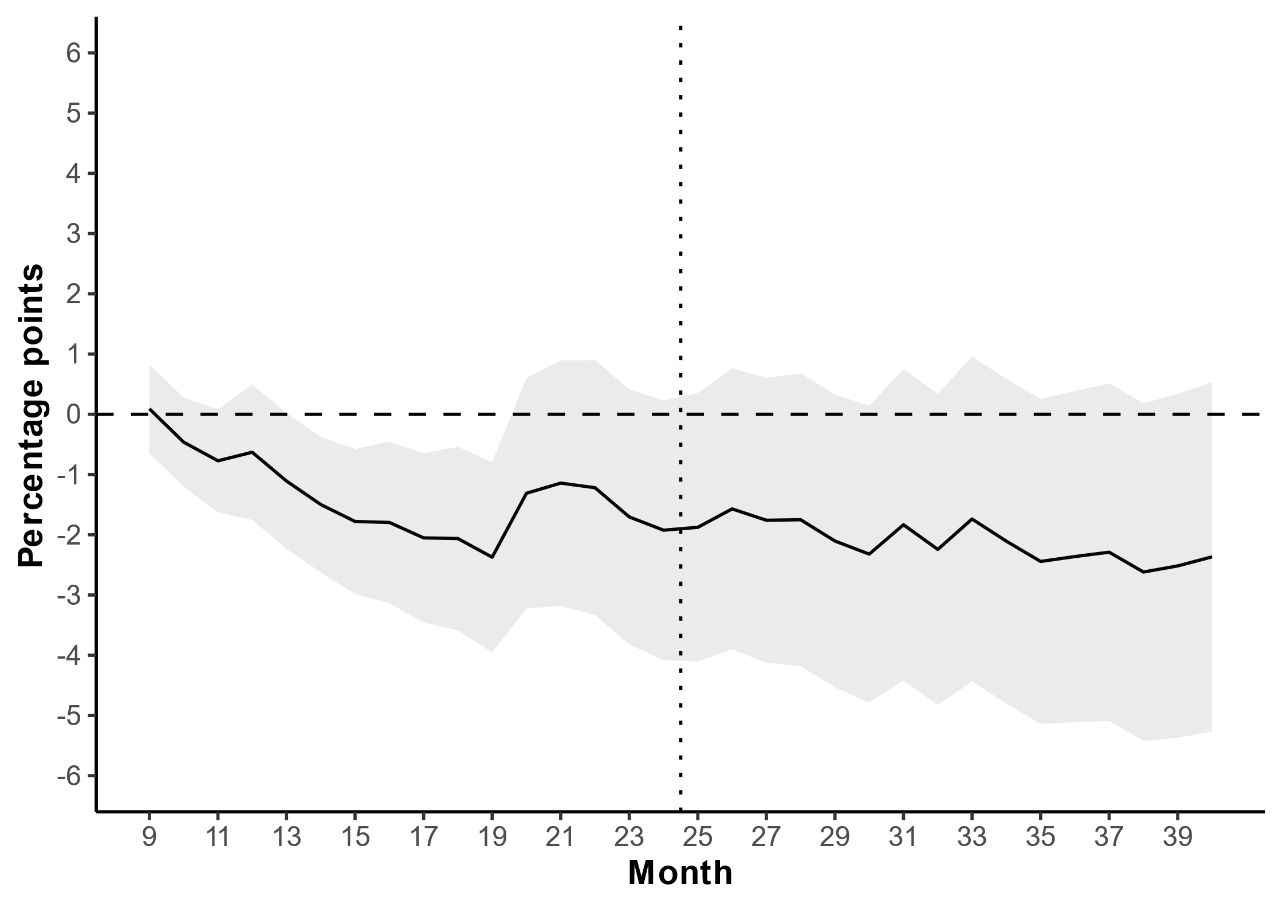


**Figure S1** Difference in the cumulative share of women who had at least one child since month 9 between basic income group and control group; 25–39-year-old women who participated in the experiment; months 9–40 with 95% confidence intervals


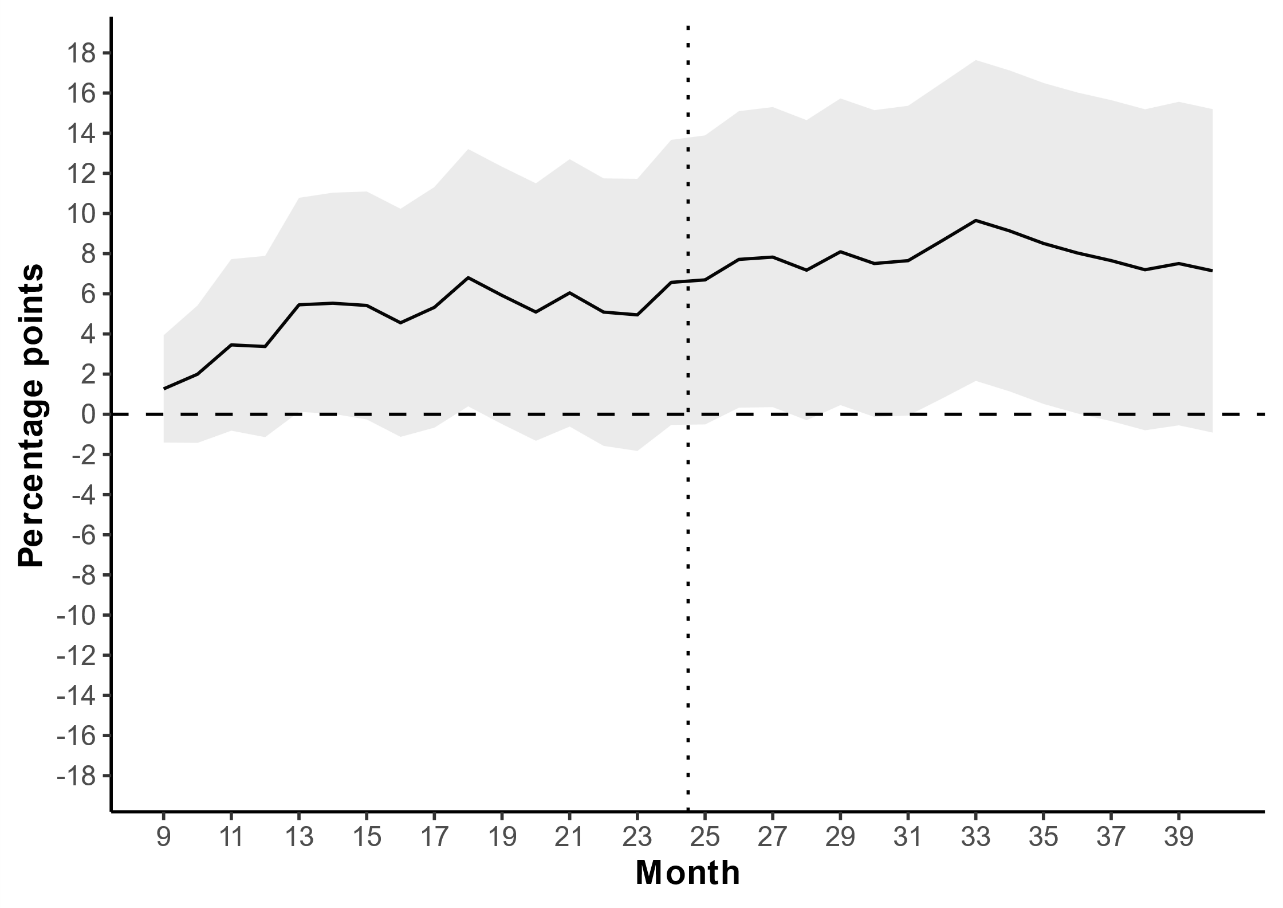


**Figure S2** Difference in the cumulative share of women who had at least one child since month 9 between basic income group and control group; 20–39-year-old women whose spouses participated in the experiment; months 9–40 with 95% confidence intervals
